# RAMBO-K: Rapid and Sensitive Removal of Background Sequences from Next Generation Sequencing Data

**DOI:** 10.1371/journal.pone.0137896

**Published:** 2015-09-17

**Authors:** Simon H. Tausch, Bernhard Y. Renard, Andreas Nitsche, Piotr Wojciech Dabrowski

**Affiliations:** 1 Centre for Biological Threats and Special Pathogens, Robert Koch Institute, 13353, Berlin, Germany; 2 Research Group Bioinformatics (NG4), Robert Koch Institute, 13353, Berlin, Germany; Seoul National University College of Medicine, REPUBLIC OF KOREA

## Abstract

**Background:**

The assembly of viral or endosymbiont genomes from Next Generation Sequencing (NGS) data is often hampered by the predominant abundance of reads originating from the host organism. These reads increase the memory and CPU time usage of the assembler and can lead to misassemblies.

**Results:**

We developed RAMBO-K (Read Assignment Method Based On K-mers), a tool which allows rapid and sensitive removal of unwanted host sequences from NGS datasets. Reaching a speed of 10 Megabases/s on 4 CPU cores and a standard hard drive, RAMBO-K is faster than any tool we tested, while showing a consistently high sensitivity and specificity across different datasets.

**Conclusions:**

RAMBO-K rapidly and reliably separates reads from different species without data preprocessing. It is suitable as a straightforward standard solution for workflows dealing with mixed datasets. Binaries and source code (java and python) are available from http://sourceforge.net/projects/rambok/.

## Introduction

The rapid developments in Next Generation Sequencing (NGS) have allowed unprecedented numbers of different organisms to be sequenced. Thanks to the output of current generation sequencing machines, viral and endosymbiont genomes can even be directly sequenced from their host since the huge amount of data generated counterbalances the presence of host sequences. However, especially de novo assembly of genomes from datasets from mixed sources is complicated by the large number of background reads, necessitating some form of pre-filtering in order to identify the relevant foreground reads (see e.g. Metcalf, Jo [1]).

Here, we present RAMBO-K, a tool which allows the rapid and sensitive extraction of one organism’s reads from a mixed dataset, thus facilitating downstream analysis.

## Implementation

**Fig 1:**
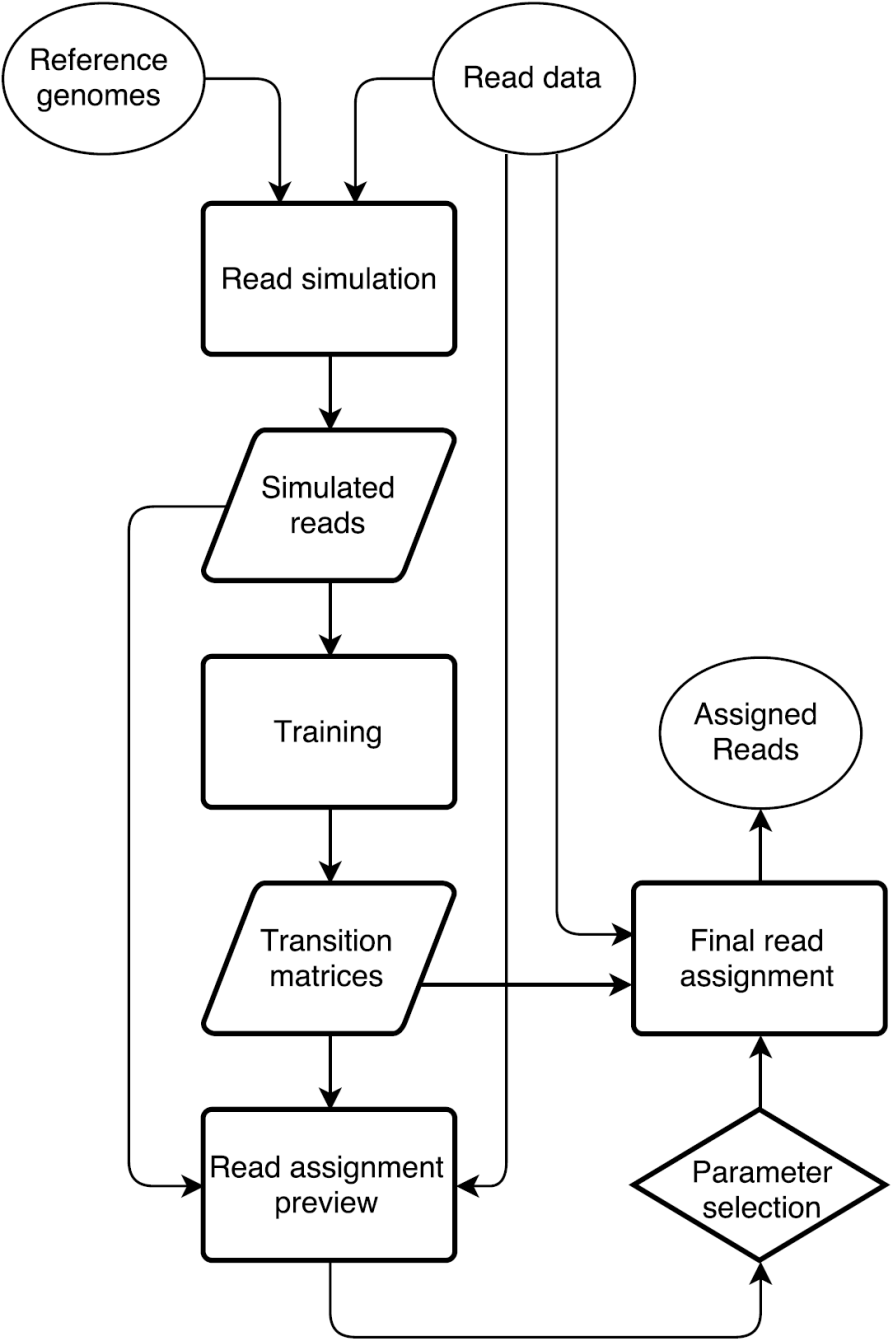
Graphical representation of RAMBO-K’s workflow. Reads are simulated from the reference genomes and used to train a foreground and background Markov chain. The simulated sequences and a subset of the real reads are assigned based on these matrices and a preview of the results is presented to the user. If this preview proves satisfactory, the same parameters are used to assign all reads.

In order to separate reads, RAMBO-K uses a reference-driven approach. The user must provide FASTA files containing sequences related to both the foreground (usually the virus or endosymbiont of interest) and the background (usually the host organism). The reference sequences do not have to represent finished genomes; collections of contigs from a draft genome or lists of sequences from different related organisms can be provided if no exact reference is known. Based on these inputs, RAMBO-K performs the sorting of reads in three steps: (i) simulation of reads from reference sequences; (ii) calculation of two Markov chains, one for the foreground and one for the background, from the simulated reads; and (iii) classification of real reads based on their conformance with the Markov chains. This workflow is visualized in Fig 1.

### Simulation of reads

It is important to ensure that the training set used for the calculation of the Markov chains is as similar to the real data set as possible. As such, in the first step the mean and the standard deviation of the read length are calculated from a user defined number of reads *n*. There is a trade-off involved in choosing the number of reads to simulate–while more simulated reads allow a better characterization of the foreground and background genomes, simulating more reads also takes more time. In our tests (data not shown), we have found 50’000 Reads to yield good results for the characterization of genomes of up to 3 Gbp while not slowing down the calculation too much. We have thus chosen 50’000 as the default value for *n*.

The *n* reads matching the length characteristics of the raw data are generated–error-free and evenly distributed–from both the foreground and the background respectively by generating n sorted random positions in each reference file. Starting from each of these positions, a string of the length of a read is read and checked for non-base characters. If no such characters are found, the characters are saved as a simulated read. The number of successfully simulated reads *m* is saved in each iteration and *n-m* reads are generated in the next iteration until a total of *n* reads have been generated. This approach has been chosen since it substitutes reading the whole reference sequence from the hard drive with a series of seek operations which speeds up the read simulation on very large reference genomes while only slightly slowing down the simulation from small reference genomes, which is fast due to the small file size. The simulation process is repeated twice to generate both a training set and a test set.

### Calculation of Markov chains

Markov chains of user-specified order k are calculated from the foreground and background read training sets: for each k-mer the observed probability of being followed by A, G, T or C is calculated. Based on these Markov chains, a score S for each read from the test set is calculated as follows:

$$S=\sum_{i=k}^{l}\text{log}\left({Pr}_{f}(B_{i}|M_{i-1})\right)-\;\sum_{i=k}^{l}\text{log}\left({Pr}_{b}(B_{i}|M_{i-1})\right)$$

where *l* is the read length, *B*_*i*_ is the base at position *i*, *M*_*i*_ is the k-mer ending at position *i* and *Pr*_*f*_ and *Pr*_*b*_ are the observed transition probabilities in the foreground and the background Markov chain, respectively. Conceptually, this is the difference in how well the read is described by the foreground and the background Markov chains. In order to avoid numeric complications which are likely to arise at higher orders, where the large number of possible k-mers leads to small observed probabilities, the logarithms of the probabilities are summed instead of multiplying the probabilities themselves [2].

**Fig 2:**
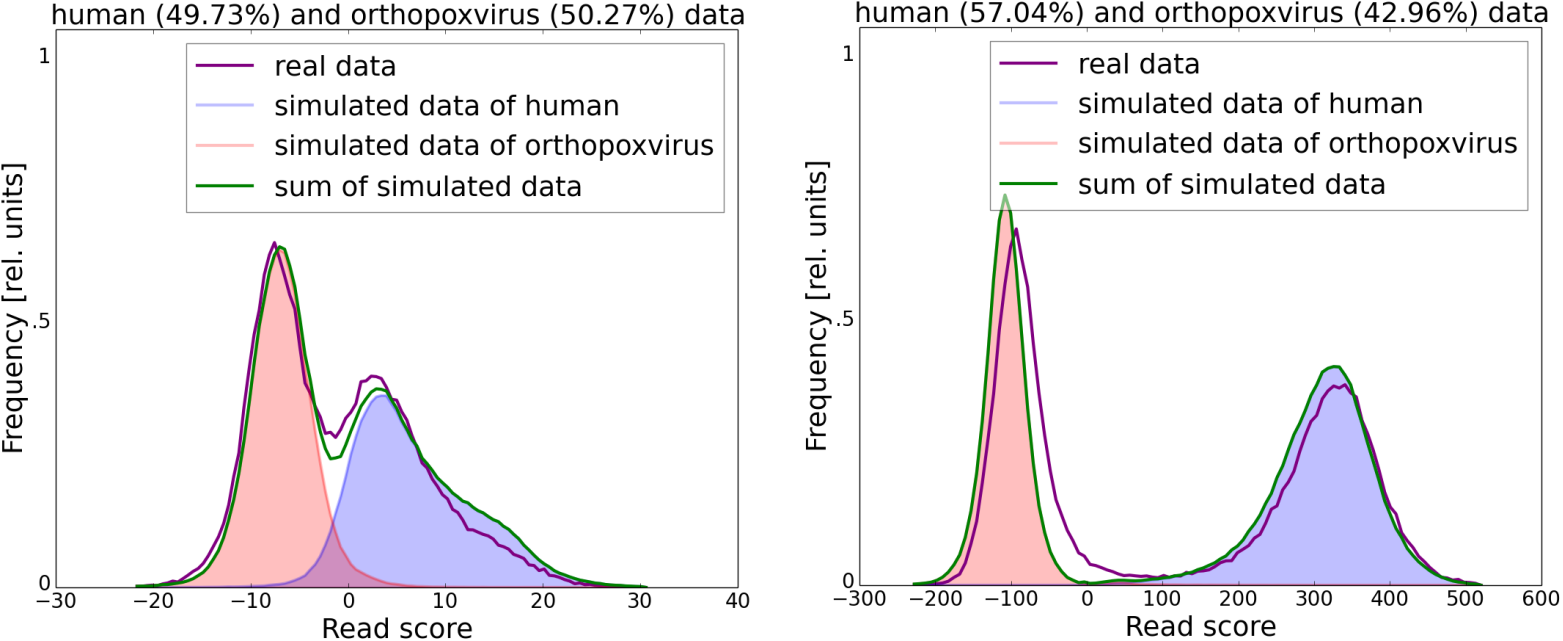
Example of the graphical output of RAMBO-K for a dataset containing human and orthopoxvirus sequences. The score distribution of both simulated and real reads is displayed for two different k-mer lengths (left: 4, right: 10), allowing the user to choose the best k-mer length and cutoff. In this case, a cutoff around -100 at a k-mer length of 10 would allow a clean separation of foreground and background reads, as visualized by the clearly separated peaks. The estimated abundance of foreground and background reads in the dataset is displayed in the figure title.

The score is also calculated for the first 50,000 reads and the scores of both test sets and the reads are then plotted. This allows the user to choose a good cutoff for the subsequent classification (Fig 2). It also allows the user to assess whether separation of the reads is likely to succeed based on the provided reference sequences. If the score distributions from the simulated data overlap well with the score distributions from the real data, as is the case in the example shown in Fig 2, the separation is likely to be successful. In such a case, the plot also gives a first overview of the dataset’s composition, since fitting the distributions of scores obtained from the test set to those from the reads allows RAMBO-K to provide a first estimation of the ratio of foreground to background reads in the data. On the other hand, a bad fit of the distribution of real and simulated read’s scores indicates a potential problem. One reason could be that the organisms present in the sample are different from the organisms whose genomes were provided as references to RAMBO-K. Often though, it can indicate a poor quality of the data and the resulting need for trimming. In S1 Fig, we have provided plots resulting from running RAMBO-K on the same dataset as used in Fig 2, but without first trimming the data.

Since the order of the Markov chain strongly influences the performance of RAMBO-K, a range of orders for which the calculation is automatically repeated can also be provided (Fig 2). Additionally, ROC plots showing the performance on the simulated data for each k are provided.

### Classification of reads

Once the user has decided upon an upper or lower cutoff and a k-mer value, RAMBO-K can be run to classify the real reads based on the previously computed Markov chains. A score is calculated for each read following the formula given in section 3.2 and a result file containing only the reads with scores below the upper or above the lower cutoff is created.

## Results and Discussion

In order to assess the usefulness of RAMBO-K, we compared its performance with that of several other tools. We used three datasets: (i) Vaccinia virus sequenced from cow lesions; (ii) Bat adenovirus sequenced from a bat, and (iii) Wolbachia endosymbiont sequenced from Drosophila. In addition to RAMBO-K, we used Kraken [3], AbundanceBin [4] and PhymmBL [2] to classify the datasets. While Bowtie2 [5] is not a classifier per se, it is often used in preprocessing to either discard all reads not mapping to the foreground reference or to discard all reads mapping to a background reference. We have included both of these mapping-based approaches in our benchmark.

The parameters for RAMBO-K on the tested datasets were selected from a range of k between four and twelve. Following the parameter estimation step described in section 3.2, the best parameter sets determined by the ROC-AUC were selected. k was consequently set to 8, 4 and 10 for the Cowpox, Bat adenovirus, and Wolbachia datasets respectively.

At the time of sequencing of the Bat adenovirus, the closest known genome was that of the distant canine adenovirus. We created our ground truth by mapping the reads to the now known Bat adenovirus genome, but gave all tools only a set of Adenovirus genomes known at the time of sequencing as references for benchmarking.

## Conclusions

As shown in Table 1, RAMBO-K is by far the fastest of all tested tools. Unlike the other tools we tested, which tend to excel either in the high sensitivity or in the low false positive rate department, RAMBO-K gives a high sensitivity at a low cost in terms of false positive assignments. Particularly when working with datasets where an exact reference is not known (such as the Bat adenovirus dataset)–which is becoming more common, especially with the expanding use of NGS in a clinical context–RAMBO-K performs better than current approaches.

**Table 1:** Benchmark results.

	Cowpox (1.3 M reads, SRS957177)				Bat adenovirus (33 K reads, SRX856705)				Wolbachia (12 M reads, SRR1508956)			
	Time[s]	SEN	FPR	F-Score	Time[s]	SEN	FPR	F-Score	Time[s]	SEN	FPR	F-Score
RAMBO-K	**31**	0.87	3.00E-04	**0.92**	**2.1**	0.79	0.05	**0.86**	**297**	1	4.00E-05	**1**
Kraken	157	0.83	2.00E-05	0.9	4.4	1	0.42	0.8	7004	0	0	N/A
AbundanceBin	20938	0	0	N/A	73	0.99	0.88	0.65	1.10E+06	0.5	0.48	0.07
PhymmBL	82556	0.68	1.00E-04	0.8	1.00E+05	0	0	N/A	1.7E+07^a^	0.5	2E-03^a^	0.64^a^
Bowtie2+	146	0.85	**1.00E-05**	0.92	5.1	0.11	**0**	0.2	419	0.99	**3.00E-06**	0.99
Bowtie2-	550	**0.95**	0.76	0.03	93	**1**	0,91	0.65	1274	**1**	0.97	0.07

The best value for each dataset is in bold. While Bowtie2+ (keeping reads mapping to the foreground reference) generally gives the lowest false-positive rate (FPR) and Bowtie2- (discarding reads mapping to the background reference) the highest sensitivity (SEN), RAMBO-K shows the best balance, providing high SEN and low FPR (F-Score) with the consistently lowest run-time. RAMBO-K outperforms other methods by the largest margin when the nearest known reference has a low identity to the sequenced genome, as in the Bat adenovirus dataset.

a: The values for PhymmBL on the Wolbachia dataset were extrapolated based on the analysis of a subset of 5% of the reads.

A large advantage of RAMBO-K for the preprocessing of NGS data lies in the graphical feedback given to the user. This allows choosing the k-mer size and cutoff best suited for each run (Fig 1). Together with its low runtime and easy installation, we believe that it represents a valuable and easy-to-implement step in the preprocessing of NGS data before assembly.

## Supporting Information

S1 Fig Example of the graphical output of RAMBO-K for an untrimmed, low-quality dataset containing human and orthopoxvirus sequences. The dataset used in this graphic is the same one as used in Fig 2 and the results for the same k-mer lengths (left: 4, right: 10) are shown. However, in this case, the reads have not been trimmed. Two effects are visible: Firstly, the distribution of the real read’s scores deviates much more strongly from the distribution of the simulated read’s scores than is the case with trimmed data. Secondly, due to this discrepancy, RAMBO-K is not able to reliably estimate the relative abundance of reads from the two organisms and the estimate varies widely between the two k-mer sizes.
(TIFF)
