# Supplementary figures and images for: RAMBO-K: Rapid and Sensitive Removal of Background Sequences from Next Generation Sequencing Data
Source: PLoS One. 2015 Sep 17;10(9):e0137896. doi: 10.1371/journal.pone.0137896 (PMC4574938; doi:10.1371/journal.pone.0137896)

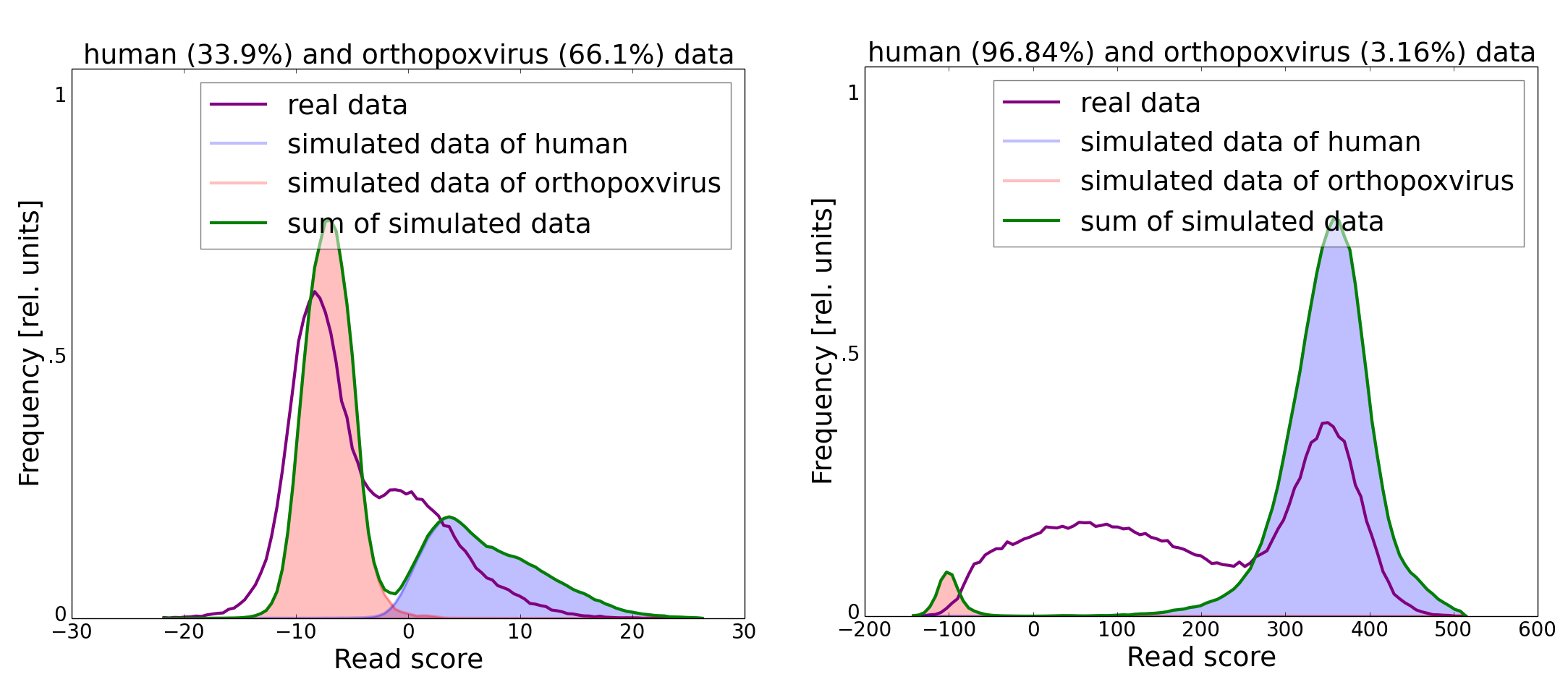

Supplement: S1 Fig — The dataset used in this graphic is the same one as used in Fig 2 and the results for the same k-mer lengths (left: 4, right: 10) are shown. However, in this case, the reads have not been trimmed. Two effects are visible: Firstly, the distribution of the real read’s scores deviates much more strongly from the distribution of the simulated read’s scores than is the case with trimmed data. Secondly, due to this discrepancy, RAMBO-K is not able to reliably estimate the relative abundance of reads from the two organisms and the estimate varies widely between the two k-mer sizes. (TIFF) [file pone.0137896.s001.tiff]
